# Effects of Pilates exercises on spine deformities and posture: a systematic review

**DOI:** 10.1186/s13102-024-00843-3

**Published:** 2024-02-22

**Authors:** Fangyi Li, Roxana Dev Omar Dev, Kim Geok Soh, Chen Wang, Yubin Yuan

**Affiliations:** Department of Sports Studies Faculty of Educational Studies, Universiti Putra Malaysia, Serdang, Malaysia

**Keywords:** Pilates, Spine deformities, Scoliosis, Kyphosis, Posture, Exercise intervention, Public health

## Abstract

**Background:**

Pilates is becoming increasingly popular amongst a wide range of people and is gaining more attention. It is also an effective means of physical rehabilitation. The aim of this systematic review is to explore the effects of Pilates on spinal deformity and posture.

**Method:**

This systematic review was conducted using four recognised academic and scientific databases (Scopus, Web of Science, PubMed and Cochrane) to identify articles that met the inclusion criteria. The secondary search used the Google Scholar and the Science Direct search engines. The search for articles for this review began in July 06, 2023 and was concluded on February 01, 2024. The search process for this study was documented using the Preferred Reporting Items for Systematic Reviews and Meta-Analyses (PRISMA 2020). The PEDro scale was used to assess the internal validity and data statistics of the studies included in this systematic review and to evaluate the quality of the studies.

**Results:**

The systematic review included nine studies that met the inclusion criteria from the 651 studies retrieved, involving a total of 643 participants. The PEDro scale scores of the studies included in this systematic review ranged from 3 to 8. The intervention was in the form of Pilates or Pilates combined exercises. The studies included in this review used outcome measures of Cobb angle, angle of trunk rotation (ATR), range of motion (ROM), chest expansion, Scoliosis Research Society Questionnaire (SRS-22r) and postural assessment. Research has shown that Pilates is effective in correcting spinal deformities and posture, as well as improving quality of life, pain relief, function and fitness.

**Conclusions:**

This systematic review provide substantial evidence that Pilates has a positive impact on improving spinal deformity and posture. However, more research is needed to validate whether Pilates can be used effectively as a physical therapy for spinal deformity rehabilitation. Pilates has considerable potential for public health interventions.

## Introduction

Any deviation from correct posture or any disturbance in postural status requires more energy and results in limited movement in the specific area where the problem occurs [1]. Body posture depends on the arrangement of individual body parts, especially the alignment of the spine [2] and pelvis [3] in the sagittal plane. Spinal deformities are abnormal curvatures or malformations of the spine that can lead to various health problems and complications [4], such as body asymmetry, muscle imbalance, loss of flexibility and back pain, as well as negative impacts on psychological aspects and quality of life [5]. These conditions can affect people of all ages, from children to the elderly, and they can be caused by various factors, including genetics, poor posture, injury or underlying medical conditions [6–8]. Spinal deformities in the form of common body posture problems mainly involve scoliosis [9], lordosis [10] and kyphosis [11]. A review of the literature produced evidence that conservative treatment, more precise application of physical therapy and various exercise regimens can be effective in modifying spinal deformity symptoms [12–16].

Physical activity has long been recognised as an essential component of physical health promotion [14]. Regular participation in physical activity helps to release psychological pressure [17], correct body posture [14], reduce chronic disease [18], maintain a healthy weight [19] and promote mental health [20]. Pilates method, created by Joseph Pilates and originally known as Contrology, was designed to achieve complete body integration and improve muscular strength, body balance and flexibility [21]. Pilates integrated the upper and lower extremities with the trunk, rather than training specific muscle groups separately [22]. With the proper selection of exercises and controlled dosing, Pilates programmes can influence the correction of postural disorders of the spinal column [16]. Tang et al. [23] showed that the Pilates exercise method was an effective physical technique for improving pain and Cobb’s angle in patients with spinal deformity. This exercise method can alleviate spinal deformities by correcting poor body posture, strengthening the muscles that influence poor body posture and maintaining body balance [24]. Pilates increases the flexibility of the spine, thus providing relief from spinal deformities [13]. However, other authors claimed that general physical therapy, including Pilates, was ineffective for correcting spinal deformities [25]. Therefore, verifying whether Pilates is an effective exercise for the correction of spinal deformities was one aim of the current review.

Pilates emphasises core strength, such as spinal strength; stretching and lengthening the spine to increase flexibility; increasing body awareness; and reducing lower back pain to help patients with spinal deformities improve posture and increase spinal stability. Jorgic et al. [26] reviewed 11 studies of Pilates on the postural status of the spine from 2010–2016, which showed that Pilates positively affects kyphosis, lordosis and scoliosis in people of all ages. Guo et al. [27] in their literature review it was mentioned that Pilates helps to reduce cobb angle and trunk rotation, relieve pain, and increase trunk ROM in patients with scoliosis, but they stated that the quality of the studies included in the literature review was not high. In their systematic review, Kuru Çolak et al. [28] stated that research on the effects of Pilates exercise on scoliosis was very limited. Previous reviews of the effects of Pilates on spinal deformities have shown a positive effect on scoliosis relief, but the studies they reviewed were not the most recent in recent years. There were limitations to previous reviews due to the number and quality of studies. As physiotherapy rehabilitation training continues to advance, more recent studies are needed to explore the effects of Pilates on alleviating spinal deformities.

The purpose of this systematic review was to examine the effects of Pilates exercise methods on the correction of spinal deformities. The study focused on how Pilates affects spinal deformity and posture. Its effects on pain relief, physical function and quality of life are also explored. The potential public health benefits of Pilates as a form of exercise will also be examined. This review covered recent studies published in English and involving patients of different ages (with symptoms including upper cross syndrome, ankylosing spondylitis (AS) and idiopathic scoliosis). Through a comprehensive review of the existing literature, the aim was to provide valuable insights into the role of Pilates in correcting spinal deformities; improving posture and quality of life; and relieving pain.

## Methodology

### Registration and protocol

This systematic review followed the Preferred Reporting Items for Systematic Reviews and Meta-Analyses (PRISMA 2020) guidelines [29] and this study was included in the International Prospective Register of Systematic Reviews ID: CRD42024509941.

### Eligibility criteria

This systematic review referenced the PICOS model in developing the inclusion and exclusion criteria for the literature, which is based on the concepts of P (Population), I (Intervention), C (Comparison), O (Outcome) and S (Study design).

Articles that met the following criteria were included in this study (See Table 1):

- Subjects must have a spinal condition (scoliosis or lordosis or kyphosis or Spine-related diseases), regardless of gender and age;
- Study design as a randomised controlled trial or quasi-experimental study;
- At least one of the trial interventions was Pilates exercise;
- Duration was not less than four weeks;
- Outcomes of the study must include the effects of Pilates exercises on the spine of the participants;
- Studies published in English.

**Table 1:** Inclusion criteria were based on the PICOS model

**PICOS**	**Inclusion criteria**
Population	spinal condition (scoliosis or lordosis or kyphosis or Spine-related diseases), regardless of gender and age
Intervention	Pilates exercise
Comparison	the experimental group was the Pilates group
Outcome	effects of Pilates exercises on the spine of the participants
Study design	randomised controlled trial (RCT) or quasi-experimental study

### Information sources

The literature search was conducted in four electronic databases (Scopus, Web of Science, PubMed and Cochrane) and two search engines (Google Scholar and the Science Direct) from 6 June 2023 to 1 February 2024 (the date the search was last conducted). The search included research articles published online.

### Search strategy

Peer-reviewed scientific published papers written in English are eligible for this systematic review. We have developed keywords based on MeSH https://www.ncbi.nlm.nih.gov/mesh/). Search terms revolve around (Pilates or Pilates exercise or Pilates training or Pilates method) and (scoliosis or lordosis or kyphosis or spinal posture or spinal deformity). After completing the first round of searches of four databases, we conducted a second literature search using search engines to ensure the comprehensiveness of the retrieved articles. We found that by searching different databases and search engines according to years not available, we noticed a spike in information about Pilates effect on spinal deformity starting in 2018. Therefore, to ensure the comprehensiveness and up-to-date nature of the studies included in this systematic review, we set the search year as 2018–2024. The search for articles for this review began in July 06, 2023 and was concluded on February 01, 2024. In addition, the research reviewers applied manual searches to examine the bibliographies in the articles included in this study and the reference lists of previous similar reviews. The retrieved articles were imported into the same database, from which duplicates were excluded; this process was undertaken by two reviewers. All the article titles and abstracts were manually checked. When disagreements arose during the search process, they were resolved by a third research reviewer to ensure that the selected papers answered the research questions posed in this review. Table 2 demonstrates the search strategy of this study and Table 3 shows the results of different databases and search engines.

**Table 2:** Keywords used for database searches

“**Pilates**”	**Operator**	“**Spinal deformity**”
Pilates OR Pilates exercise OR Pilates training OR Pilates method	AND	scoliosis OR lordosis OR kyphosis OR spinal posture OR spinal deformity

**Table 3:** Search strategy for all databases and search engines

Databases	Search strategy	Results	Search date
Scopus (2018–2024)	TITLE-ABS-KEY: ("Pilates" OR "Pilates exercise" OR "Pilates training" OR "Pilates method" AND "scoliosis" OR "lordosis" OR "kyphosis" OR "spinal posture" OR “spinal deformity”)	7	06.07.2023–02.01.2024
Web of Science (2018–2024)	((TS = Pilates or Pilates exercise or Pilates method or Pilates training)) AND TS = (scoliosis or lordosis or kyphosis or spinal posture or spinal deformity))	112	06.07.2023–02.01.2024
PubMed (2018–2024)	((Pilates [Title/Abstract] OR Pilates exercise [Title/Abstract] OR Pilates training [Title/Abstract] OR Pilates method [Title/Abstract]) AND (scoliosis [Title/Abstract] OR lordosis [Title/Abstract] OR kyphosis [Title/Abstract] OR spinal posture [Title/Abstract] OR spinal deformity [Title/Abstract]))	210	06.07.2023–02.01.2024
Cochrane (2018–2024)	In Trials; TITLE-ABS-KEY: Pilates or Pilates exercise or Pilates training or Pilates method AND TITLE-ABS-KEY:scoliosis or lordosis or kyphosis or spinal posture or spinal deformity	19	06.07.2023–02.01.2024
Science Direct (2018–2023)	Pilates or Pilates exercise or Pilates training or Pilates method and scoliosis or lordosis or kyphosis or spinal posture or spinal deformity	12	06.07.2023–02.01.2024
Google Scholar	Pilates or Pilates exercise or Pilates training or Pilates method and scoliosis or lordosis or kyphosis or spinal posture or spinal deformity and trials	291	06.07.2023–02.01.2024

### Selection and data collection process

Two reviewers (F,L and C, W) independently screened the retrieved documents against the inclusion and exclusion criteria. Created a new folder containing all retrieved articles. A first screening was performed based on article titles and abstracts, and a database was created to include articles that met the requirements of this review. The second screening was done by reading the full text of the articles in the database to determine which articles could be included in this systematic review. When this process encountered disagreements, the third reviewer (R, D) provided input.

### Data extraction

Upon completion of the data search, data eligible for the study was retrieved according to the following format: (a). name of the author, nationality and year of publication of the article; (b). research trial design; (c). characteristics of the experimental intervention; (d). characteristics of the study population; and (e). research outcome. Data consistent with the study was extracted into a standardised schema, and a database was created by one reviewer and checked by two other reviewers.

### Quality assessment and addressing the risk of bias

This review examined the quality of the experiments using the Physiotherapy Evidence Database (PEDro) scale. This scale has proven to be a valuable standard for assessing experimental quality during the construction of systematic reviews. The purpose of using the PEDro scale in this systematic review was to assess the intra-experimental validity of the studies included in this review and sufficient data statistics to judge the quality of the studies based on the scores. It contains 11 criteria to evaluate the internal and external validity of an experiment. Criterion 1 evaluates external validity but is not included in the overall PEDro score out of 10. Criteria 2–9 are used to evaluate the internal validity of the experiment, whereas criteria 10–11 focus on evaluating whether the results of the experiment were analysed using valid statistics. The quality of each experiment was assessed by one reviewer, and the assessment criteria were yes (1 point) and no (0 points) for statistical purposes. The PEDro score for an article usually ranges from 0–10. The higher the PEDro score, the better the experimental research methodology used in the experiment; that is, the approach was more appropriate. Studies with a PEDro score between 8 and 10 were rated as methodologically excellent in terms of quality; those with a score between 5 and 7 were of good quality; those with a score between 3 and 4 were of moderate quality; and those with a score of less than 3 were of low quality [30].

The Cochrane Risk of Bias (RoB 2.0) tool was used to assess the risk of bias for each study. Risk of bias was assessed for bias in the randomisation process, bias due to deviation from the intervention, bias due to missing results, bias in the measurement of the data, and bias in the choice of reported outcomes [31]. The internal effect risk of bias assessment for each study was completed independently by two reviewers (F.L and R.D) and recorded as: low-risk, unclear and high-risk and marked with different colour-coding systems. A third reviewer (K.S) checked the results and resolved disagreements.

### Data synthesis

Results were not pooled due to the varied methods, data, and contexts of these studies. Instead, we provided a narrative synthesis of the results of the included studies (See Table 5). The interventions we identified were Pilates or Pilates combined with exercise. Participants were identified as populations with spinal deformity disorders (e.g., scoliosis, adolescent idiopathic scoliosis, or AS) or with body posture problems. We identified that the study outcomes included important factors that affect spinal alignment (Cobb angle, ATR, Chest Expansion and ROM etc.). The summary table was provided participant’s age, gender, subgroup details, intervention duration, intervention frequency, experimental design and outcomes.

## Results

### Search results

**Fig. 1:**
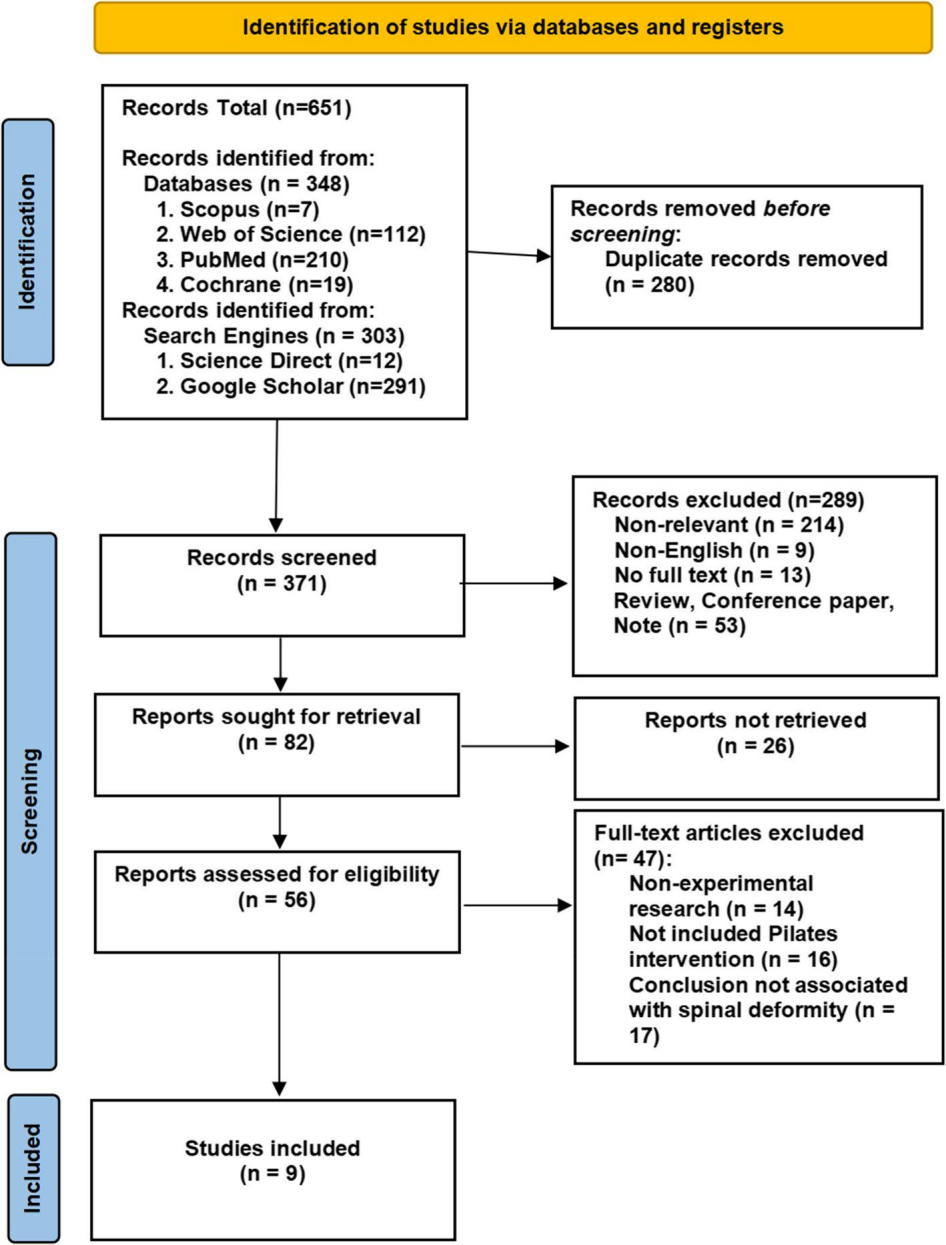
Preferred Reporting Items for Systematic Reviews and Meta-analysis (PRISMA 2020) flow diagram of the systematic review screening process

Figure 1 illustrates the retrieval workflow for this study. A total of 9 studies were included out of 651 records retrieved from different databases and search engines. Of the retrieved records, 280 were excluded as duplicates. Duplicate articles were excluded using the EndNote 20 citation management system. After removing duplicates, 371 articles were eliminated by assessing the article titles and abstracts. The remaining 56 articles were reviewed and 47 of these were eliminated, leaving 9 articles that met the inclusion criteria. These articles came from different countries and global regions, including Spain (*n* = 2), Turkey (*n* = 2), Brazil (*n* = 1), Iran (*n* = 2), Kosovo (*n* = 1) and Egypt (*n* = 1). Publication occurred in the following years: 2020 (*n* = 3), 2021 (*n* = 1), 2022 (*n* = 3) and 2023 (*n* = 2).

### Study quality assessment

The PEDro scale was used in this study to assess the research quality of the included articles (Table 4). The 9 articles were scored from 3 to 8 using the PEDro scale. All the studies scored on the baseline comparability criterion. Overall, 77.8% (*n* = 7) of the studies used randomised groups. Due to the more specialised nature of Pilates exercises and the assessment of spinal deformities, a review by a specialised therapist was required. Few studies scored on the scale for criteria 6 and 7. Nonetheless, all the authors strove to ensure that all the patients received treatment. Some studies indicated that appropriate follow-up work would be undertaken to obtain additional data.

**Table 4:** PEDro score

Author	Criteria											Total score items 2-11/10
	1	2	3	4	5	6	7	8	9	10	11	
Cibinello, F. U (2020) [32]	1	1	1	1	1	0	0	0	0	1	1	6
Rrecaj-Malaj, S (2020) [33]	1	1	0	1	0	0	0	1	1	0	1	5
Başaran Özden, C (2022) [34]	1	1	1	1	0	0	0	0	0	1	1	5
Ozturk, N (2022) [35]	1	0	0	1	0	0	0	0	0	1	1	3
González-Gálvez, N (2023) [36]	1	1	0	1	0	0	0	1	1	1	1	6
Ahmadi, F. (2021) [37]	1	0	0	1	0	0	0	0	1	1	0	3
Karkousha, R. (2023) [38]	1	1	0	1	0	1	1	0	1	1	1	7
Gandomi, F. (2022) [39]	1	1	1	1	0	0	1	1	1	1	1	8
González-Gálvez, N (2020) [40]	1	1	0	1	0	0	0	0	0	1	1	4

Item score: 1, meets criteria; 0, does not meet criteria; Criteria: 1, Eligibility criteria; 2, Random allocation; 3,Concealed allocation; 4, Baseline comparability; 5, Blind subjects; 6, Blind therapists; 7, Blind assessors; 8, Adequate Follow-up; 9,Intention to Treat Analysis; 10, Between group comparisons; 11, Point estimates and variability

In terms of PEDro scale scores, the average score of the literature included in this systematic review was 5.2/10. The overall quality of the literature was moderate. Of the nine studies included, five were RCT, three were Non-RCT, and one was a randomised clinical trial. As the participant populations of the 5 RCT studies suffered from different types of spinal disorders, including thoracic hyperkyphosis, upper cross syndrome and AS. The overlapping intervals of their interventions were small, which could have led to statistical heterogeneity due to the diversity of intervention effects. Therefore, in conjunction with the above reasons, this systematic review was not meta-analysed.

### Population characteristics

An assessment was conducted of the population characteristics (see Table 5) of the 9 studies included in the review. (1) Sample size: the total sample size of the 9 studies was 643 subjects; the smallest of these studies involved 15 subjects and the largest included 236. (2) Gender: gender was not specified in three studies. Two studies included only female participants; the remaining four included both males and females. In the studies where gender was specified, there were 316 female subjects and 181 males overall. (3) Age: the subjects ranged in age from 5 to 58. One study focused on children aged 5–6 years; six studies focused on children and adolescents aged 8–30 years; and one study focused on adults aged 28–58 years. One study did not specify age. In addition to these three characteristics, the subjects were either school students or patients suffering from spinal deformities.

**Table 5:** Population, intervention and outcome

**Author**	**Population**	**Intervention**						**Outcome**
		**Age**	**Sex**	**Type**	**Duration**	**Frequency**	**Study design**	
Cibinello, (2020) [32]	*N* = 40, school children, PG: *n* = 20, CG: *n* = 20	8–12	unknow	Mat Pilates	12 weeks	2 times/week 50min per time	RCT	Posture →
Rrecaj-Malaj, (2020) [33]	*N* = 69, Cobb angle between 10° ~ 45°	10–17	25M 44FM	Combined Schroth and Pilates Exercises	24 weeks	60 min/day	Non-RCT	Cobb angle↑, ATR↑, Chest Expansion↑, Trunk Flexion↑, QoL↑
Başaran, Özden (2022) [34]	*N* = 34, with adolescent idiopathic scoliosis PG: *n* = 16, CG: *n* = 18	15–30	5M 29FM	Pilates	8 weeks	2 times/week 60min per time	RCT(S)	Spine Deformity, QoL, and perception of deformity in scoliosis → Back and Low Back Pain↑ Posture↑
Ozturk, N (2022) [35]	*N* = 66, preschool children, PG: *n* = 31, CG: *n* = 35	5–6	unknow	Pilates	10 weeks	2 times/week	Non-RCT	Posture↑ Physical fitness↑
González-Gálvez, (2023) [36]	*N* = 103, adolescents with thoracic hyperkyphosis, PG: *n* = 52, CG: *n* = 51	13.48 ± 1.23	27M 76FM	Pilates	38 weeks	2 times/week 15min per time	RCT	Thoracic Kyphosis in Relaxed Standing Position↑ Hamstring Extensibility↑
Ahmadi, F. (2021) [37]	*N* = 15, female students of University ormitories with hyperkyphosis and hyperlordosis	unknow	15FM	Pilates	10 weeks	2 times/week 90min per time	Non-RCT	Thoracic Kyphosis↑ Lordosis Malformations↑
Karkousha, R. (2023) [38]	*N* = 40, female with the upper cross syndrome, EG: *n* = 20, CG: *n* = 20	17–22	40FM	Pilates	4 weeks	2 times/week	RCT	Spinal Curvature↑, Balance, Function↑, Pain↑
Gandomi, F. (2022) [39]	*N* = 40, with ankylosing spondylitis, APG: *n* = 12, ASG: *n* = 14, CG: *n* = 14	28–56	unknow	Aqua Stretch Aqua Pilates	6 weeks	4 times/week 60min per time	RCT	Pain↑, function↑, QoL↑, Spinal ROM↑
González-Gálvez, (2020) [40]	*N* = 236, high school student, EG: *n* = 118, CG: *n* = 118	13.15 ± 1.24	124M 112FM	Pilates	36 weeks	2 times/week 15min per time	RCT	Averted the increase of the thoracic curvature, and decreased the curvature of the lumbar lordosis and pelvic tilt in standing position; avoided a greater increase of thoracic curvature in active alignment in standing position; and avoided the increase of thoracic curvature in trunk fexion

*EG* Experimental Group, *PG* Pilates Group, *CG* Control Group, *APG* Aqua Pilates Group, *ASG* Aqua Stretch Group, *RCT* Randomised Controlled Trials, *RCT(S)* Randomised Clinical Trials, *ATR* Angle of Trunk Rotation, *QoL* Quality of Life, *DCF* deep cervical fexors, *ROM* range of motion, *CROM* cervical range of motion, *M* Male, *FM* Female

### Intervention characteristics

The intervention characteristics of the 9 studies, as presented in Table 5, include the type of intervention, intervention duration and training frequency. In terms of intervention type, all the studies used Pilates as the main form of intervention, with one using mat Pilates and another using aquatic Pilates. Two studies used a format that compared Pilates with other interventions. One study combined Schroth and Pilates exercises and one study compared Aqua Pilates to Aqua Stretch.

The duration of the Pilates intervention in most studies ranged from eight to 12 weeks. The shortest intervention duration was four weeks, followed by six weeks, while the longest study intervention duration was 38 weeks and one studies lasted 36 weeks.

In terms of frequency of intervention, seven studies used a frequency of two times per week, with two studies lasting 15 min per intervention, one study lasting 50 min per intervention, one study lasting 60 min per intervention and one study lasting 90 min per intervention. Two studies did not specify the duration of each intervention. One study was conducted four times per week for 60 min each time. Another study was conducted for 60 min per day.

### Outcomes

Nine studies (see Table 5) with Pilates as the independent variable of the intervention modality with a dependent variable related to spinal deformity were included in this review. A total of 643 subjects ranging from 5 to 58 years of age were involved. Most studies focused on the effects of Pilates on spinal deformity and posture. Some studies also investigated the effect of Pilates on hamstring extensibility, while others investigated the effects of Pilates on quality of life, pain, functionality and physical fitness, in addition to focusing on spinal deformity and posture.

#### Effects of Pilates on spinal deformity and posture

The effects of Pilates on thoracic kyphosis was mentioned in 2 study outcomes. The results obtained by González-Gálvez [36] showed significant gains in terms of sagittal spinal curvatures, thoracic kyphosis in a relaxed standing position and hamstring extensibility in adolescents after 36–38 weeks of Pilates training. Ahmadi’s [37] study of female university students also demonstrated that Pilates training was effective in improving thoracic kyphosis and lordosis malformations in female undergraduates.

The effects of Pilates on trunk flexion was mentioned in 2 research outcomes. Rrecaj-Malaj [33] reported that combined Schroth and Pilates exercises benefited the Cobb angle, Angle of Trunk Rotation (ATR), chest expansion and trunk flexion in adolescents with mild and moderate idiopathic scoliosis. The results of another González-Gálvez [40] study indicated that adolescents participating in Pilates training avoided an increase in thoracic spine curvature in the standing position; reduced the curvature of the anterior lumbar spine and pelvic tilt; avoided a further increase in thoracic spine curvature in active alignment in the standing position; and avoided an increase in thoracic spine curvature in the anterior tilt of the trunk.

The effects of Pilates on spinal curvature and spinal range of motion were mentioned in two other studies. Karkousha’s [38] study of a group of women with upper cross syndrome showed that Pilates not only had a significant effect on the spinal curvature of the patients but also improved their balance function. Gandomi [39] compared the effects of Aqua Stretch and Aqua Pilates on spine posture in patients with AS. The results showed a significant improvement in spinal range of motion in both groups.

Three studies have mentioned the effects of Pilates on posture. A study [34] of patients with adolescent idiopathic scoliosis showed no significant effects on spine deformity or perception of deformity in scoliosis after eight weeks of Pilates training, however there was a significant improvement in participants’ posture. In Ozturk’s [35] study involving 5–6 year-old preschool children, the PG showed significant improvements in rounded shoulders and thoracic kyphosis after Pilates training, relative to the CG. On the other hand, Cibinello [32] showed that after 12 weeks of mat Pilates training, the postures of some subjects improved because the latter were at a stage of rapid skeletal development and body structure growth. Therefore, it is impossible to determine whether this was a result of the Pilates intervention.

#### Effects of Pilates on quality of life, pain, function and physical fitness

Table 5 shows that five studies addressed the effects of Pilates on quality of life, pain, function and physical fitness. Three studies mentioned the positive impact of Pilates on quality of life, which can be achieved by alleviating the distress associated with spinal deformities. Rrecaj-Malaj [33] indicated that real idiopathic scoliosis causes deformity of the spinal column, which directly affects a patient’s quality of life. The patients’ self-image and quality of life were enhanced by a combined Schroth and Pilates exercise intervention. Gandomi [39] assessed how Aqua Stretching and Pilates improved the quality of life and function of patients with AS. Both interventions had significant effects on quality-of-life improvement, which was higher in the Aqua Stretch group. However, Başaran Özden [34] conducted eight weeks of Pilates training with adolescent idiopathic scoliosis patients aged 15–30 years, finding that it did not improve their quality of life but it did reduce their back and lower back pain, as well as significantly improve their posture. Karkousha’s [38] study demonstrated the effectiveness of Pilates exercises versus traditional physiotherapy in relieving pain and improving function in patients with upper cross syndrome. In a study of 5–6 year-old preschool children, not only were their head, shoulder and spine postures assessed, but they were also tested for physical fitness (using scores from the Flamingo balance, sit and reach, standing broad jump, 30-s sit-up, bent arm hang, and 20-m shuttle run tests) [35]. The results showed that Pilates exercises both enhanced the children’s posture and positively impacted their physical fitness.

## Discussion

The 9 studies included in this review involved people of different ages, predominantly adolescents but also children and adults. The interventions were all Pilates-based; although the findings they assessed varied, they all highlighted common themes. Pilates is a mind–body exercise that focuses on core stability, flexibility, posture control and breathing [41], and it has recently become a target of interest as a beneficial form of exercise [42]. The findings of this review provide strong evidence that muscle strength and control can be enhanced to correct spinal deformities and improve posture through Pilates training, and that participants’ self-image and social interaction can also be enhanced through Pilates. The study results show that Pilates can be used to benefit a variety of people of different ages, including children, junior high school students, high school students, college students, people with AS and those with upper cross syndrome. The findings of this systematic review have important implications for the development of targeted Pilates training programmes for postural correction, improving spinal deformities, and the promotion of physical health and well-being.

### Effects of Pilates on spinal deformity and posture

Recent research and clinical trial results have shown that Pilates has emerged as a promising intervention for correcting spinal deformities and improving posture. This systematic review provides a comprehensive discussion centred on the background and findings of recent relevant studies with the aim of revealing whether Pilates has positive effects on spinal deformity and posture. Exploring the potential mechanisms and clinical treatment outcomes of these effects will inform and provide insights for subsequent relevant research and practice by academics and healthcare professionals.

The diagnosis of scoliosis is based on a standing anteroposterior radiograph with a Cobb angle greater than 10° [43]. In addition to Cobb’s Crossing, the Angle of Trunk Rotation (ATR) is another important indicator of scoliosis. Two studies in the literature included in this systematic review addressed the effect of the Cobb angle and ATR on scoliosis. Rrecaj-Malaj’s [33] study showed that both groups of patients significantly improved in terms of lordosis malformations while wearing and not wearing a brace for the Cobb angle (from 21.97 ± 4.99° to 18.11 ± 6.39°; from 14.19 ± 3.11° to 11.66 ± 2.73°), ATR (from 7.19 ± 1.36° to 5.36 ± 1.66°; from 4.72 ± 1.04° to 3.58 ± 0.94°), chest expansion (from 2.56 ± 0.84 cm to 3.46 ± 0.72 cm; from 2.57 ± 0.87 cm to 3.52 ± 0.72 cm), trunk flexion (from 9.55 ± 1.95 cm to 14.33 ± 2.40 cm; from 9.82 ± 2.61 cm to 13.98 ± 2.18 cm). These results were achieved in 69 patients aged 11–17 years with mild-to-moderate idiopathic scoliosis after 24 weeks of combined Schroth and Pilates exercises. The results of this study are consistent with those of previous related studies [24, 44]. However, Başaran Özden [34] compared the Cobb angle and ATR in the PG and CG groups in their study at baseline and after eight weeks of intervention, finding no significant changes in either parameter in patients with adolescent idiopathic scoliosis after the Pilates intervention. Based on our discussion, it was concluded that the inconsistency between the results of these two studies may have been because Schroth exercises were superior to Pilates exercises in improving the Cobb angle and ATR.

Spinal curvature is the criterion for determining thoracic kyphosis and lordosis malformations [45]. From the literature included in this review, seven studies involved spinal curvature. Ahmadi’s [37] study on female students showed that through Pilates exercise intervention, students improved on average by 13.68 degrees of hyperkyphosis and 10.79 degrees of hyperlordosis. Weight, height and body mass index were effective factors in alleviating thoracic or lumbar spine abnormalities. Thus, Pilates practice based on the scientific principles of therapeutic planning can be effective in correcting kyphosis and lordosis malformations in female students. The research data gathered by Karkousha [38] showed that participants in the EG and CG groups pre-treatment had mean ± SD of spinal curvature of 45.9 ± 2.6 and 45.2 ± 2.2 degrees, respectively, while the measurements post-treatment were 42.1 ± 2.7 and 35.1 ± 2.4 degrees, respectively. The result confirmed that the Pilates exercise programme proved better than a traditional physical therapy programme in improving spinal curvature in those with upper cross syndrome. In Gandomi’s [39] study comparing the effects of Aqua Pilates and Aqua Stretch exercises on spinal posture in patients with AS, the results for each group of subjects revealed significant improvements in elevated spinal curvature and spinal ROM. The results of González-Gálvez’s [36] data showed a significant reduction in the thoracic kyphosis angle and lumbar lordosis angle in the relaxed standing position for the PG. The adjusted mean difference found between the groups for the thoracic curve was 5.9°. The results of two other studies by González-Gálvez [40] examined how the Pilates method improved the sagittal spinal curvatures and hamstring extensibility of adolescents. Based on the findings, with prolonged Pilates training, adolescents can effectively alleviate increased thoracic spine curvature in the standing position; avoid a further increase in thoracic spine curvature during active alignment in the standing position; and avoid an increased thoracic spine curvature during the anterior trunk tilt. Furthermore, in Rrecaj-Malaj’s [33] study of adolescents with idiopathic scoliosis, spine curvature was only measured at baseline and not further interpreted. The results of the studies discussed above indicate that Pilates intervention has significant effects in terms of reducing spinal curvature in different populations and different patients.

Posture problems can negatively impact overall health, leading to diseases, pain or functional disorder and thus potentially affecting quality of life in both childhood and adulthood [46]. Spinal deformity is a common postural problem. This systematic review discusses the effects of Pilates on spinal deformities while incidentally understanding its effects on posture. González-Gálvez [40] showed that with prolonged Pilates intervention training, lumbar lordosis and pelvic tilt curvature can be reduced, thereby decreasing the prevalence of anterior pelvic tilt in the adolescent population. Ozturk [35] conducted a 10-week Pilates intervention with 66 preschoolers aged 5–6 years old and assessed their body posture, which included the head, shoulders, back, lower back and legs. The results showed significant effects on rounded shoulders and thoracic kyphosis in the PG relative to the CG group after Pilates training. On the other hand, Cibinello [32] showed that after 12 weeks of mat Pilates training, some postures improved. However, since the subjects were at a stage of rapid skeletal development and body structure growth, it was impossible to determine whether this improvement resulted from the Pilates intervention. The findings of these studies are consistent with previous research showing that Pilates leads to notable improvements in posture. Pilates can be promoted as an effective intervention to improve posture in people of all ages.

Overall, the above studies provide strong, compelling and valuable evidence for the role of Pilates in improving spinal deformity and posture. These recent results have demonstrated that Pilates can be used by people of different ages and with different spinal disorders. Underlying postural problems can also be alleviated through Pilates interventions. These findings again demonstrate the breadth of the Pilates audience and the effectiveness of this type of workout. They also highlight the potential of Pilates as an effective and enjoyable exercise in the field of public health and wellness. Combining these benefits and integrating Pilates into clinical practice and public health campaigns would be a cost-effective strategy for promoting physical activity and preventing postural deformities.

### Effects of Pilates on quality of life, pain, function and physical fitness

The evidence from recent studies and research shows not only that Pilates exercises excel in correcting spinal deformities and improving posture, but also that people who participate in Pilates exercises experience positive impacts on their quality of life in terms of pain, functioning and physical fitness. Of the studies included in this systematic review, five articles mentioned the positive effects of Pilates. This part of the discussion comprehensively analyses the impacts of Pilates on quality of life, pain, function and physical fitness. Exploring the potential mechanisms and clinical treatment effects of these effects will inform and provide insights for subsequent relevant research and practice by academics and healthcare professionals.

Three of the studies mentioned the impact of Pilates on quality of life, with such exercises having the capacity to alleviate the distress associated with spinal deformities. Rrecaj-Malaj [33] indicated that real idiopathic scoliosis causes deformity of the spinal column, directly affecting a patient’s quality of life. In this study, the quality of life level of all the subjects was tested using the SRS-22r (Scoliosis Research Society Questionnaire). The results showed that the patients’ self-image and quality of life were enhanced by a combined Schroth and Pilates Exercises intervention. Gandomi [39] used the ASQOF (Ankylosing Spondylitis Quality of Life) questionnaire [47] in their study to assess the effects of Aqua Stretch and Aqua Pilates exercises on the quality of life of people with AS. The results showed that the subjects’ quality of life significantly improved. The effects were greater on the water stretching group than the water Pilates group, as shown by the MDC (Minimal Detectable Change) and MCID (Minimal Clinically Important Difference) values. However, this did not prevent the authors from recommending water exercises as part of a treatment plan for AS patients. However, Başaran Özden [34] conducted eight weeks of Pilates training with adolescent idiopathic scoliosis patients aged 15–30 years. At the end of the intervention, the Scoliosis Research Society-23 (SRS-23) questionnaire was used to survey the quality of life of the subjects, with the results showing that their quality of life had not improved.

Three articles covered the effects of Pilates on pain relief. Pilates was found not to affect quality of life in adolescent idiopathic scoliosis patients, but the intervention effectively reduced their back and lower back pain and significantly improved their posture [34]. Karkousha’s [38] study with the Visual Analogue Scale (VAS) demonstrated the effectiveness of Pilates exercises versus traditional physiotherapy in relieving pain in patients with upper cross syndrome. Both Aqua Stretch and Aqua Pilates can provide significant pain relief for those with AS and have great potential to improve their condition [37]. These studies provide strong evidence that Pilates has great potential to relieve the pain caused by spinal deformities or postural disorders.

In terms of improved functionality, Gandomi [39] evaluated physical function using the Bath Ankylosing Spondylitis Functional Index (BASFI) and the 40-m walking test (40-MWT). Improvements were seen in both the Aqua Stretch and Aqua Pilates groups, with no significant difference between the two groups. The authors explained that the warmth of the water, the use of comfortable and painless exercises, and the hydrostatic pressure in the water reduced the time taken to complete the 40-MWT. Similarly, one study provided preliminary evidence that Pilates was superior to traditional physiotherapy in improving balance function [38].

In a study of 5–6 year-old preschool children, not only were the children’s head, shoulder and spine postures assessed, but they were also tested for physical fitness (using scores from the flamingo balance, sit and reach, standing broad jump, 30-s sit-up, bent arm hang and 20-m shuttle run tests) [35]. The results showed that Pilates exercises both enhance children’s posture and positively impact their physical fitness.

Overall, recent research and clinical trials have provided valuable indications of the ways in which Pilates improves quality of life, relieves pain, improves function and enhances physical fitness. Pilates interventions have shown promise in improving quality of life, relieving pain and improving function. However, in terms of enhancing physical fitness, the paucity of the available evidence is too great to make firm conclusions, and more research is needed to further substantiate this. These findings demonstrate the potential of Pilates to enhance people’s lives and improve their sense of well-being. Incorporating Pilates into public exercise and wellness programmes could provide individual participants with more innovative, fun and enjoyable physical activities, thus enriching their leisure time. Pilates is a potentially valuable sport for improving people’s standard of living and sense of well-being.

### Implications for public health

The findings of this systematic review reveal that Pilates can be applied to a wide range of people; that it is effective in correcting spinal deformities, improving posture, enhancing quality of life and relieving pain; and that such exercises have considerable potential in terms of their public health benefits. Pilates is a low-cost, easy and high-reward form of exercise that can positively impact public health in several ways. It could be incorporated into public health exercise programmes as a valuable form of physical activity that promotes good health and improves people’s standard of living.

Pilates improves people’s physical fitness, which would have an important impact on public health. Pilates emphasises core strength, flexibility and overall body conditioning [48]. Regular and disciplined Pilates practice builds muscular strength, endurance and flexibility, thus helping to reduce the risk of injuries, especially those related to poor posture and muscle imbalance [49]. This systematic review study found that Pilates can improve spinal deformity and poor posture by increasing spinal strength in patients, a result consistent with those of previous studies. Such findings offer a valuable rationale for utilising Pilates to enhance public health and particularly the well-being of patients suffering from spine-like disorders and/or poor posture.

Another advantage of Pilates is that it relieves pain from chronic conditions, especially back and lower back pain. Evidence suggests that many people have relieved their chronic lower back pain and reduced their dependence on painkillers by participating in a Pilates exercise programme [50]. For sedentary, healthy middle-aged women, a Pilates exercise programme can improve the respiratory system, relieve shoulder and lower back pain, as well as enhance parameters such as shoulder girdle, abdominal strength and endurance, and hip and shoulder mobility [51]. The findings of these studies are consistent with those of this review study [34, 38, 39]. When included in a public health exercise programme, Pilates appears to be a physical activity that brings guaranteed benefits for people with chronic pain, as well as a promising therapeutic tool for physical rehabilitators, rehabilitation coaches and healthcare professionals.

Pilates helps to improve the mental health of participants, which in turn improves their social connectedness and sense of community. Pilates improves not only people’s physical health but also their mental health [52]. Regular Pilates practice can reduce stress, anxiety and depression, thus contributing to better overall mental well-being. Therefore, treatment with the Pilates method should be encouraged so that a better quality of life can be achieved [53]. Group Pilates classes build social connections and a sense of community, which can have positive impacts on mental health and overall well-being. Social support is a proven key factor in public health outcomes. Pilates, with its novel teaching style, relaxed workout atmosphere and enjoyable workout process, provides participants with a friendly platform for communication when they are exercising. It also provides opportunities for more community participants to be physically active, which can increase community cohesion and people’s sense of well-being, thus providing opportunities to build a network of social support platforms.

In conclusion, the public health impact of Pilates is considerable. Due to its accessibility, Pilates is a low-intensity exercise that can be used in public health programmes, and the populations in which such activities could be conducted are unlimited. Therefore, there is a strong need to build partnerships with community organisations, physical rehabilitation facilities, healthcare professionals and educational institutions when integrating public health exercise programmes. This would ensure the universality and breadth of the exercise population, maintain the sustainability of Pilates interventions and promote knowledge exchange and innovation. Whilst Pilates exercise can make a positive public health contribution, it should form part of a wider approach to safeguarding the health of the population. Encouraging and facilitating physical activity such as Pilates should be one of many strategies to promote physical health in the general population. Certainly, more pilot studies are needed to provide guidance and explanations for the effectiveness and practice of Pilates in the public health context.

### Limitations and future research

There are limitations to this systematic review that must be acknowledged. Firstly, only 9 articles were included in the review. There may have been some flaws in the inclusion criteria and search strategy developed to screen the articles, resulting in the exclusion of some articles that met the requirements. In future review studies, a more comprehensive search strategy and finer-grained inclusion criteria need to be developed to ensure a universal and more authoritative representation of the relevant articles.

Secondly, the sample sizes of the articles included in this review study varied considerably, with the largest being 236 and the smallest 15. This meant the authors were unable to explore potential bias, which could have affected each set of results. Future review studies could focus on research conducted using large sample sizes and standardised intervention trials to provide more precise results and findings.

Thirdly, the studies included in this systematic review used Pilates exercises of varying intensity, which prevented a more precise definition of the parameters by which Pilates affects spinal deformity. Future studies could focus on research with long intervention durations and similar intervention intensities to obtain more precise trial parameters.

Finally, the articles included in this systematic review rarely used hidden allocation and blinded assessment, and they had fewer follow-up studies. In future studies, long experimental designs are necessary and extended follow-up periods are important to determine the effectiveness of the interventions.

## Conclusion

The findings of this systematic review provide substantial evidence that Pilates has a positive impact on improving spinal deformity and posture. Patients can improve spinal muscle strength to correct spinal posture by participating in Pilates training. Pilates has also been shown to have a significant effect in lowering the Cobb angle, decreasing the angle of trunk rotation and increasing spinal range of motion. Pilates is a highly promising exercise that has the advantages of being reliably safe, unrestricted in terms of training space and suitable for people of all ages. Patients can relieve pain and improve their quality of life, physical functioning and fitness by participating in Pilates training. However, the limited number of articles included in this review and the uncertainty over the standard of their quality mean that caution is required when interpreting and applying the results of this review in subsequent relevant research and practice. Therefore, more generalisable and high-quality follow-up studies are needed to explore this area further.

Considering the benefits of Pilates, it can form an excellent backup to a public health exercise programme. Pilates is a low-cost, easy-to-perform and highly rewarding exercise. Incorporating it into public health exercise programmes and making it accessible to a wider population can be an effective way to increase the population’s motivation to be physically active, thereby improving their standard of living and quality of life. Further research is needed to better understand the causal effects of Pilates on public health and its potential as an intervention method for enhancing public health.

## Data Availability

The dataset supporting the conclusions of this article is included within the article.
